# *In silico* characterization of the gating and selectivity mechanism of the human TPC2 cation channel

**DOI:** 10.1085/jgp.202313506

**Published:** 2025-02-21

**Authors:** Alp Tegin Şahin, Ulrich Zachariae

**Affiliations:** 1 Computational Biology, School of Life Sciences, University of Dundee, Dundee, UK; 2 School of Medicine, University of St. Andrews, St. Andrews, UK; 3 Biological Chemistry and Drug Discovery, School of Life Sciences, University of Dundee, Dundee, UK

## Abstract

Two-pore channels (TPCs) are twofold symmetric endolysosomal cation channels forming important drug targets, especially for antiviral drugs. They are activated by calcium, ligand binding, and membrane voltage, and to date, they are the only ion channels shown to alter their ion selectivity depending on the type of bound ligand. However, despite their importance, ligand activation of TPCs and the molecular mechanisms underlying their ion selectivity are still poorly understood. Here, we set out to elucidate the mechanistic basis for the ion selectivity of human TPC2 (hTPC2) and the molecular mechanism of ligand-induced channel activation by the lipid PI_(3,5)_P_2_. We performed all-atom *in silico* electrophysiology simulations to study Na^+^ and Ca^2+^ permeation across full-length hTPC2 on the timescale of ion conduction and investigated the conformational changes induced by the presence or absence of bound PI_(3,5)_P_2_. Our findings reveal that hTPC2 adopts distinct conformations depending on the presence of PI_(3,5)_P_2_ and elucidate the allosteric transition pathways between these structures. Additionally, we examined the permeation mechanism, solvation states, and binding sites of ions during ion permeation through the pore. The results of our simulations explain the experimental observation that hTPC2 is more selective for Na^+^ over Ca^2+^ ions in the presence of PI_(3,5)_P_2_*via* a multilayer selectivity mechanism. Importantly, mutations in the selectivity filter region of hTPC2 maintain cation conduction but change the ion selectivity of hTPC2 drastically.

## Teaser

By molecular dynamics simulations and *in silico* electrophysiology, Şahin and Zachariae show how the lipid PIP2 opens the human TPC2 ion channel and that its ion selectivity is established by an interplay of key structural elements of the pore.

## Introduction

Ion channels are essential components of all cells. They form membrane-integral proteins that mediate the efficient and often selective exchange of ions across lipid membranes, which are otherwise impermeable to ions. Their function is key to maintaining ionic homeostasis in the cell and its compartments, as well as constituting one of several ways for a cell to communicate with its surroundings (Ackerman and Clapham, 1997). Inside eukaryotic cells, organelles employ similar principles to regulate intracellular ion exchange and ion-based signal transduction, facilitated by the function of organellar and vesicular ion channels.

Two-pore channels (TPCs) form an important channel family found primarily in endolysosomal membranes (Patel, 2015). Endolysosomes are membrane-enclosed organelles, which play a key role in cell survival and cell death. They are also crucial for viral entry into and release from eukaryotic host cells (Afghah et al., 2020). TPCs thus represent prime targets for therapeutic agents against viruses such as Ebola and SARS-coronaviruses. In the case of Ebola, TPCs have been validated as a major target for the development of antiviral drugs and drug design efforts are presently underway (Sakurai et al., 2015).

TPCs, comprising the subfamilies TPC1–3 (Patel, 2015; Rahman et al., 2014), are ancient members of the cation-selective, voltage-gated ion channel superfamily, sharing structural similarities with other types of eukaryotic Na^+^- and Ca^2+^-selective channels such as transient receptor potential (TRP) channels. They are believed to represent an evolutionary transition stage from the four-domain monomeric to the homo-tetrameric, more complex voltage-gated ion channels (Chen et al., 2021). In accordance with their organellar location, encoding genes of TPCs can be found in eukaryotes but not prokaryotes (Shah et al., 2022). TPC1 is more abundant in endolysosomal organelles while TPC2 is preferentially localized to late endosomes and lysosomes (Ruas et al., 2014). TPC3 is not expressed in humans, but mammalian and vertebrate orthologs can be found both in endolysosomal and plasma membranes (Rahman et al., 2014; Ruas et al., 2014).

**Figure 1.**
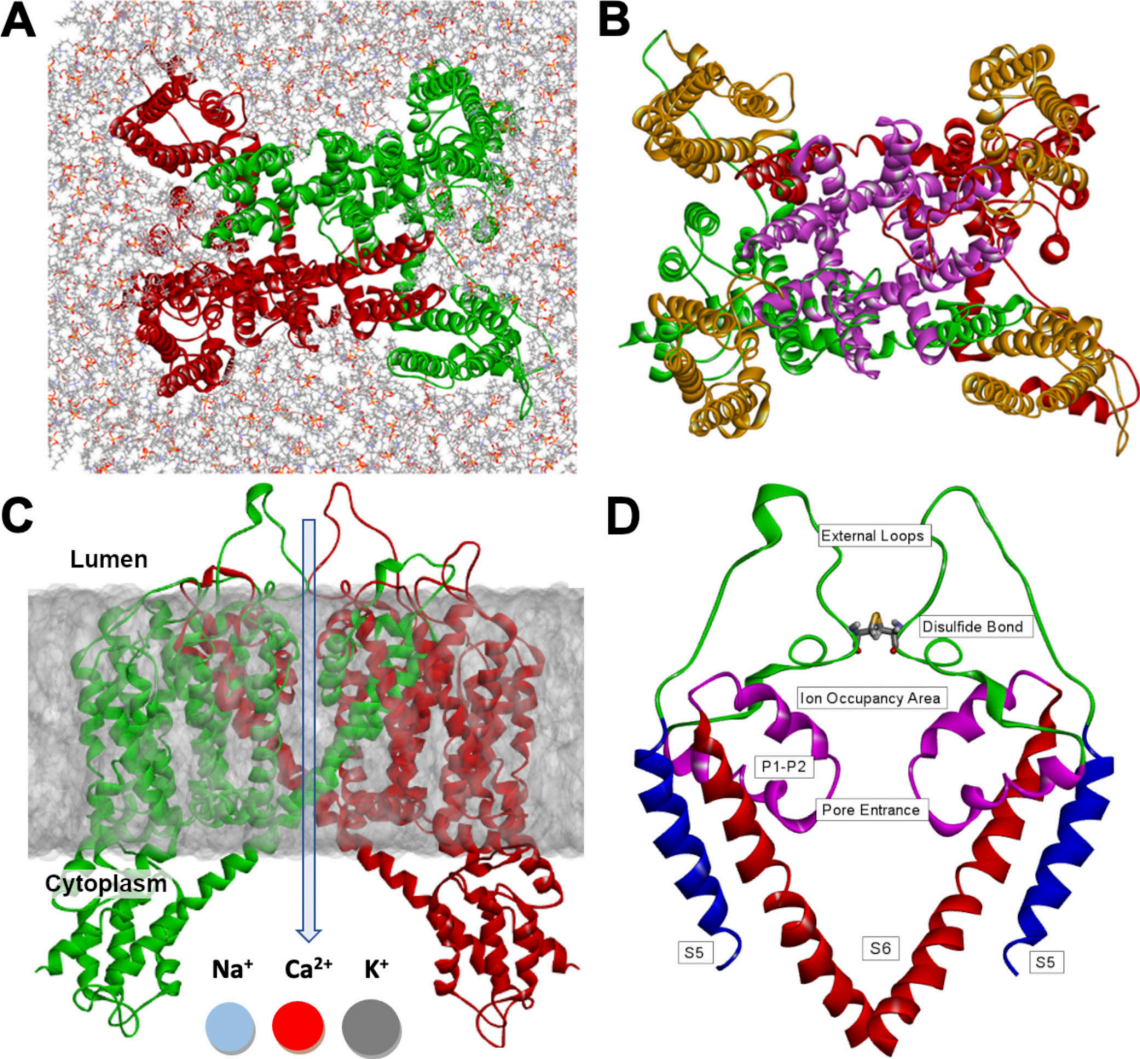
**(A)** Top view of the dimeric structure of hTPC2 in a POPC membrane; the two subunits are shown in red and green. **(B)** The pore-forming domain (purple) and voltage-sensing domains (orange) are highlighted within the two subunits of hTPC2. **(C)** Location of hTPC2 in the endolysosomal membrane and direction of ion flux from the endolysosomal lumen to the cytosol. **(D)** Schematic of the pore architecture of hTPC2. The panel shows a slice through the pore domain; the inner pore is formed by helices S6 from 6-TM-I and 6-TM-2 of each subunit, and the selectivity filter (pore entrance) is located in a loop section between the pore helices P1 and P2 from 6-TM-I and 6-TM-2 of each subunit.

In the endolysosomal membrane, TPCs form homodimers. Despite their variety and the existence of different isoforms, the overall structure of TPCs is highly conserved (Guo et al., 2016). Each subunit is comprised of two homologous Shaker-like six-transmembrane (6-TM) repeat domains (termed D1 and D2 or 6-TM-I and 6-TM-II; Fig. 1 A) (Kintzer and Stroud, 2018, She et al., 2019). A single chain of TPCs thus contains two voltage-sensing domains (VSDs) formed by helices I-S1-S4 and II-S1-S4, as well as two pore-forming domains composed of helices I-S5-S6 and II-S5-S6 (Fig. 1, B–D). The existence of two pore-forming domains per subunit has given rise to the nomenclature of this channel family (Brailoiu et al., 2010; Kintzer and Stroud, 2018). In addition to their TM domains, TPCs possess EF-hand motifs on the cytosolic side, which are generally known as Ca^2+^ recruitment sites (Lewit-Bentley and Réty, 2000). As opposed to plant TPC1, the mammalian TPCs’ EF-hands however lack the essential negatively charged residues, and as a result, Ca^2+^ is unlikely to bind to mammalian-type EF-hand motifs in TPCs (Kintzer and Stroud, 2016). Finally, the subunits comprise a C-terminal domain and an N-terminal domain that have been reported to play a functionally important role in channel activation. Deletion of these domains prevents channel activity, but their mechanistic importance and role in the channels’ ion selectivity have yet to be determined (Kintzer and Stroud, 2018).

Channels of the TPC family can be gated by both voltage stimuli and a range of different ligands (Pitt et al., 2010). In particular, the channels respond to the binding of nicotinic acid adenine dinucleotide phosphate (NAADP) and the lipid PI_(3,5)_P_2_ (phosphatidylinositol-3,5-bisphosphate; PIP2) (Brailoiu et al., 2010; She et al., 2019), alongside electrochemical or voltage gradients. They are permeable to the cations Na^+^, Ca^2+^, and K^+^, each with different permeability levels (Fig. 1 C) (Guo et al., 2017). TPC2 is known to be largely voltage-insensitive and activated mainly by ligand binding. In the PIP2-bound state, human TPC2 (hTPC2) is highly selective for Na^+^ compared with Ca^2+^ with a permeability ratio of P_Na_:P_Ca_ ≈ 10:1 in whole lysosomal recordings and P_Na_:P_Ca_ ≈ 17:1 in plasma membrane recordings (She et al., 2019). In contrast, the activation of TPC1 is strongly dependent on both voltage and ligands (Brailoiu et al., 2010; Pitt et al., 2010; She et al., 2018).

PIP2 trafficking regulates a wide range of cellular processes and is especially associated with endolysosomal functions. In the case of hTPC2, the binding of PIP2 alone is sufficient to activate the channels, with a variety of studies showing that PIP2 binding predominantly triggers Na^+^ flux through TPCs (Wang et al., 2012). Notably, recent studies have demonstrated that in the presence of NAADP, TPCs can change their ion selectivity from a preference for Na^+^ to Ca^2+^. This means that to date, TPCs are the only ion channels reported to change their ion selectivity in response to ligand binding (Brailoiu et al., 2009, 2010; Pitt et al., 2010; She et al., 2019; Wang et al., 2012, 2012). NAADP is a potent and well-known Ca^2+^-mobilizing second messenger, which initiates Ca^2+^ release from endolysosomal organelles (Shah et al., 2022). Recent research on the NAADP regulation of TPCs has shown that NAADP likely does not directly bind to the TPCs but via binding proteins such as Lsm12 (Zhang et al., 2021; Saito et al., 2023).

**Figure 2.**
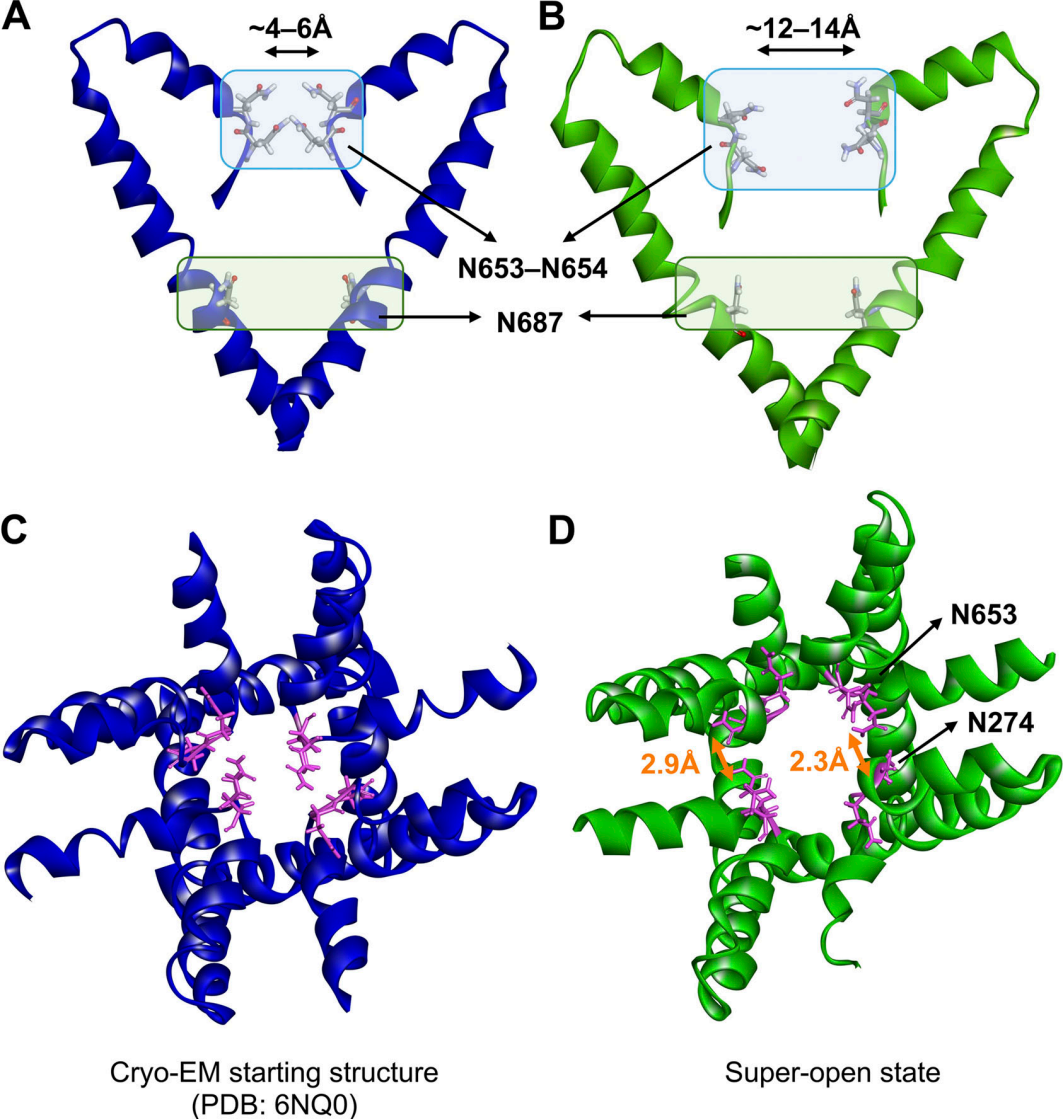
**Conformation of the key asparagine residues in the SF. (A)** Cryo-EM structure (PDB: 6NQ0). **(B)** Super-open state was observed in the simulations. **(C and D)** Reorientation of the key SF residues (N653 and N274, magenta) from the cryo-EM open (blue) to the super-open state (green) with H-bond formation between the asparagine side chains (distances shown in orange).

So far, the selectivity and ligand-gating mechanisms of hTPC2 have been insufficiently understood. The selectivity filter (SF) sequences of hTPC2 are _270_TTANN_274_ (filter I) and _651_VVNNW_655_ (filter II) (Guo et al., 2017) from each subunit. The hTPC2 cryo-EM structure shows that the SF residues are located at the entrance of the pore region (Fig. 2) (She et al., 2019). These residues are conserved between the Na^+^-selective human and mouse TPC1–2 but not in the non-selective plant TPC1 (Guo et al., 2017). Recently, Guo et al. reported that mutations of V652 and N653 in filter II substantially affected the selectivity of hTPC2 for Na^+^, whereas mutation of A272 only had a subtle effect, indicating that the residues on filter II may be the main determinants of TPC selectivity (Gunaratne et al., 2023; Guo et al., 2017). In that study, the selectivity for Na^+^ versus Ca^2+^ conduction was measured for WT and triple mutants interchanging between the SF motifs of hTPC2 and the non-selective plant channel AtTPC1. Furthermore, molecular dynamics (MD) simulations of TPC1 and TPC2 have demonstrated the importance of hydrophobic gating and the difference in overall protein flexibility between the apo and the holo (lipid-bound) channel states (Milenkovic et al., 2021). Additionally, they highlighted the role of the central channel cavity in Na^+^ interactions (Milenkovic et al., 2021).

Here, we set out to elucidate the mechanistic basis for the ion selectivity of hTPC2 and the molecular mechanism of ligand-induced channel activation by PIP2. We used *in silico* electrophysiology simulations to study Na^+^ and Ca^2+^ permeation across hTPC2 and investigated the conformational changes related to pore gating induced by the presence or absence of PIP2. Our findings show that the PIP2 interaction with the VSDs is allosterically linked to conformational changes in the SF as well as the cytoplasmic bundle crossing (hydrophobic gate). According to our simulations, ion selectivity arises from a multilayered mechanism comprising the luminal channel entrance, the SF, and the central channel cavity, which often serves as a reservoir for more than two Na^+^ ions and enables a combined overflow and distant knock-on mechanism to enhance Na^+^ conduction rates.

## Materials and methods

### MD simulations and in silico electrophysiology

**Figure S1.**
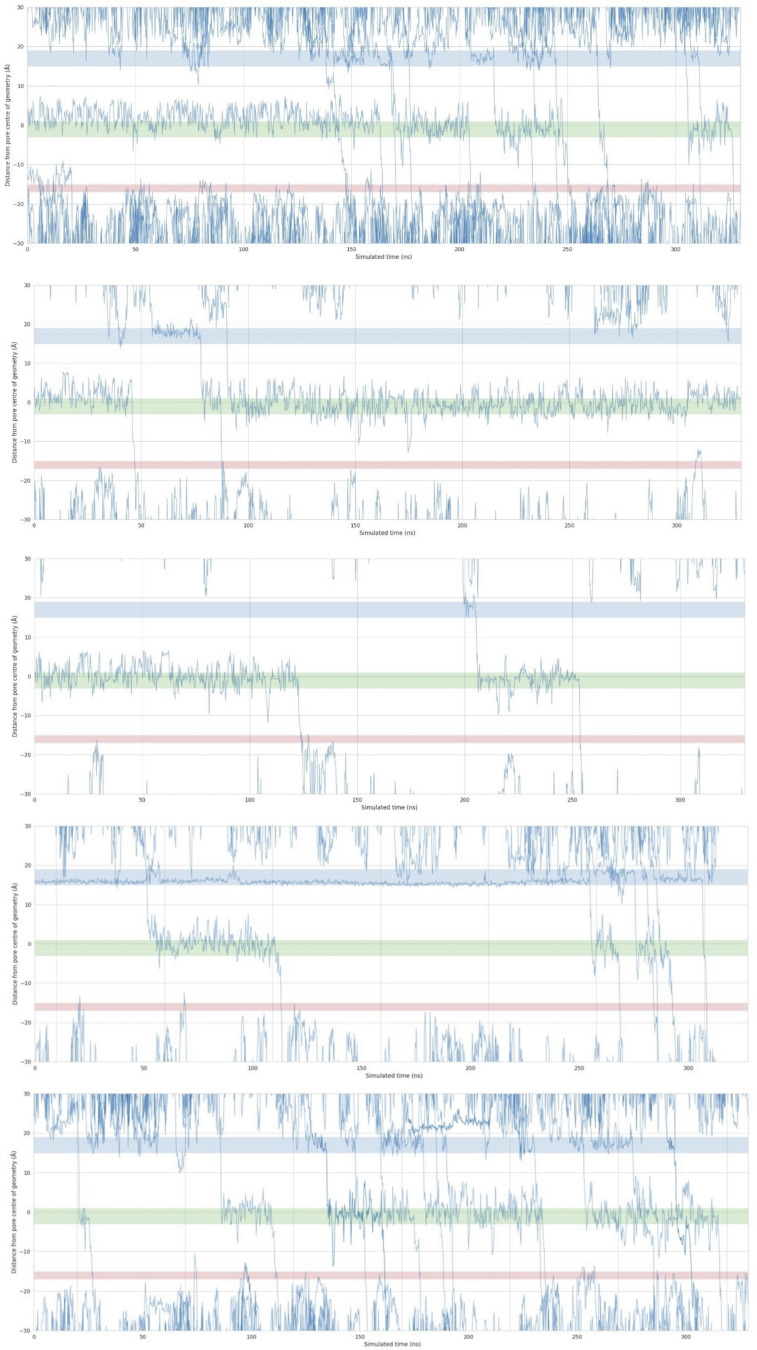
**Na** ^**+**^ **ion permeation traces for simulations in LysM, demonstrating that ion permeation exhibits no significant lipid dependence in the simulations.**

Our simulations were based on the recently published cryo-EM structure of the PIP2-bound state of hTPC2, annotated as the open state of the channel (PDB ID: 6NQ0) (She et al., 2019). hTPC2 was embedded into 1-palmitoyl-2-oleoyl-sn-glycerol-3-phosphocholine (POPC) membranes. In some test simulations, we also used mammalian lysosomal membranes (LysM) to probe for a potential lipid dependence of ion conduction, which we did not observe (see Table S1 and Fig. S1 for Na^+^ permeation traces). The bilayer systems were generated using the CHARMM-GUI server (Jo et al., 2008). The initial box had a dimension of 150 Å in both the x- and y-direction and 145 Å in the z-direction. The protein was aligned in the bilayer using the PPM server (Jo et al., 2008; Lomize et al., 2012).

Unless otherwise stated, we carried out five repeat simulations for each different setup and simulation condition investigated. In each condition, the system was equilibrated initially for 2 ns, then a 20-ns preproduction equilibration without membrane voltage was performed, and following this, different lengths of production runs (between 250-ns and 1-μs duration each), with or without membrane voltage were carried out (see Table S2 for a summary of all simulations). The pH-dependent simulations were conducted under protonation states reflecting pH 3.5 and pH 4.5 conditions on the lysosomal face, wherein only the acidic residues facing the lysosomal side of the protein changed protonation states.

Membrane voltages were generated using an applied external electric field yielding voltages of −70, −200, −350, −700, and +700 mV, respectively (Aksimentiev and Schulten, 2005). After obtaining the super-open state of the hTPC2 SF in four out of the initial set of five simulations with bound PIP2, we used the super-open state for all following simulations under voltage, as this state corresponded to an ion-conductive conformation. To examine the ion selectivity of hTPC2 in aqueous solutions, we used either 0.6 M NaCl or 0.6 M CaCl_2_ in monocationic solutions, or a mixture of 0.3 M NaCl and 0.3 M CaCl_2_ in dicationic solutions, together with the TIP3P model for water molecules (Jorgensen and Tirado-Rives, 2005). Ions and water were added with GROMACS2022, which was also used for all simulations (Van Der Spoel et al., 2005). The CHARMM36m force field was used for the protein, ions other than Ca^2+^, and lipids (Huang et al., 2017). For the Ca^2+^ ions, the multisite model developed by Zhang et al. was employed, which has recently been shown by us and other groups to reliably model Ca^2+^ currents in a range of ion channels (Ives et al., 2023; Liu et al., 2023; Schackert et al., 2023; Zhang et al., 2020). For the investigation of the pore structure, architecture, and radius profile, the CHAP program was used (Klesse et al., 2019). The LINCS algorithm was utilized to constrain bond lengths involving hydrogen atoms (Hess et al., 1997).

Hydrogen mass re-partitioning (HMR) was used (Gao et al., 2021) together with a 2-fs time step for all simulations reported in the Results section (Table S2). Harmonic distance restraints were applied between the α-carbon atoms of residues R316 and E695 to prevent dewetting of the hydrophobic gate at the cytoplasmic exit of the hTPC2 channel in long time-scale simulations of ion conduction started from the super-open state. An earlier set of simulations with mono-cationic Na^+^ ions using HMR and a 4-fs time step without distance restraints displayed frequent dewetting of the hydrophobic gate, especially at lower voltages, affecting ion conduction through the pore (see Table S2). No distance restraints were used for the simulation and analysis of the ligand-dependent channel opening dynamics.

The systems were simulated in the NpT ensemble, where the temperature was maintained at T = 310 K using the Nosé-Hoover thermostat (Evans and Holian, 1985), and the pressure was maintained semi-isotropically at 1 bar using the Parrinello–Rahman barostat (Parrinello and Rahman, 1981).

### Simulation analysis: Principal component analysis (PCA) and State-Specific Information (SSI)

The PCA of the spontaneous channel opening transition, which yields the most ion-conductive conformation of hTPC2, was performed with GROMACS 2022 utilities using a set of simulation trajectories reflecting closed states without bound PIP_2_ (total length: 1.7 µs) and the open and super-open (highly conductive) states obtained with PIP_2_ (total length: 1.63 µs) under voltage. After obtaining the PCA eigenvectors from the entire set of trajectories, the individual simulation trajectories of hTPC2 in the fully open (PIP_2_) and closed states (no PIP_2_), respectively, were projected back onto the space spanned by PCA eigenvectors 1 and 2.

Our PCA analysis showed a long-distance correlated opening motion which included the VSDs, where the PIP2 binding sites are located, and the SF of hTPC2. To identify the residues that form the allosteric pathway between the distal PIP2 binding site and the SF, we used a mutual-information–based analysis (SSI), which we recently developed and employed to decipher allosteric pathways in G protein-coupled receptors (Vögele et al., 2025; Thomson et al., 2021, *Preprint*; Thomson and Zachariae, 2025). Briefly, SSI highlights conformational state changes in protein residues that are highly correlated with the change in an “external” switch variable, in this case, the presence or absence of bound PIP2. This is achieved by determining the mutual information between the conformational change and the change in the external variable.

### Simulation analysis: Ion permeation, solvation, and conduction cooperativity

We used in-house Python scripts to determine ion permeation pathways, the number of permeation events, ion coordination numbers, and the mutual information (SSI) between binding site occupancy changes (available from https://figshare.com/articles/journal_contribution/Tpc_analysis_scripts/28264301). For the ion conduction SSI, we followed the excess SSI approach previously described in more detail in Ives et al. (2023). PENSA was used to calculate the ion density maps (Vögele et al., 2025).

### Online supplemental material

**Figure S2.**
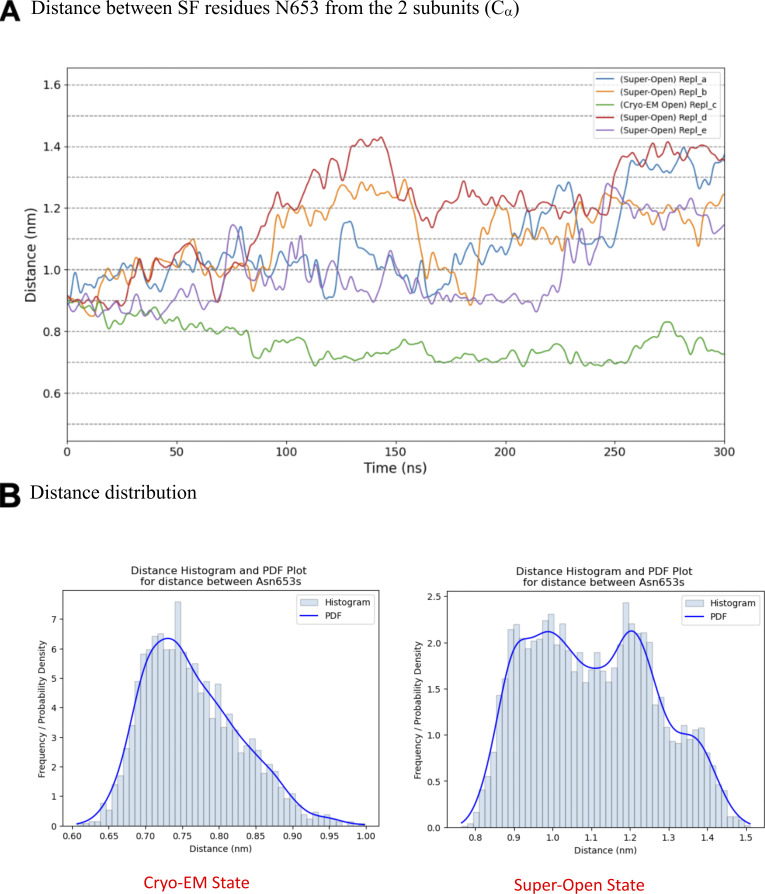
**Distance between SF residues N653 from the two subunits (C_α_).** **(A and B)** The development of the super-open state of the hTPC2 SF with SF-diameter-over-time plots (A) and probability distribution histograms and functions (PDFs) compared with the cryo-EM state (B).

**Figure S3.**
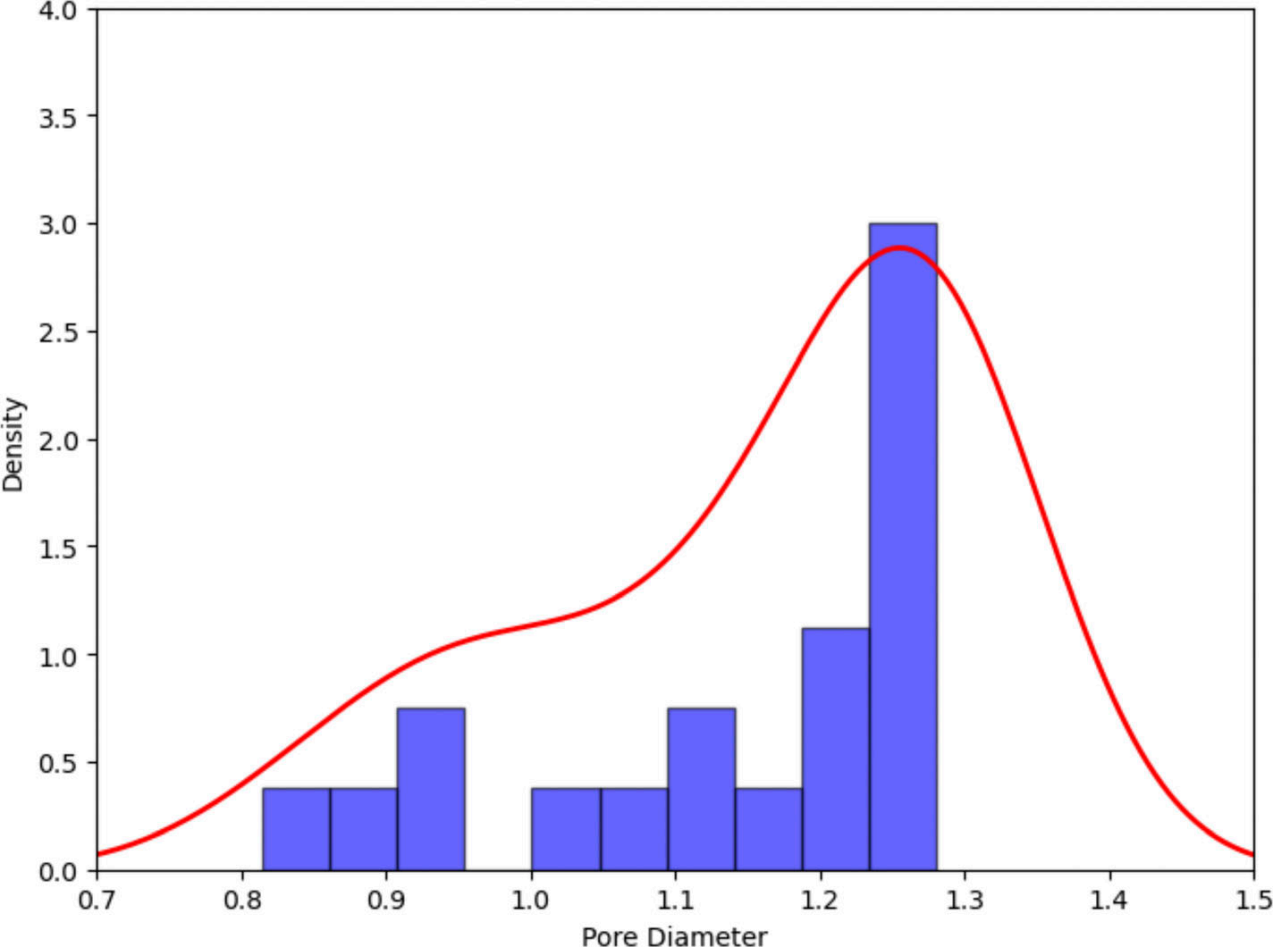
The rate of permeation events versus SF diameter at a voltage of −350 mV.

**Figure S4.**
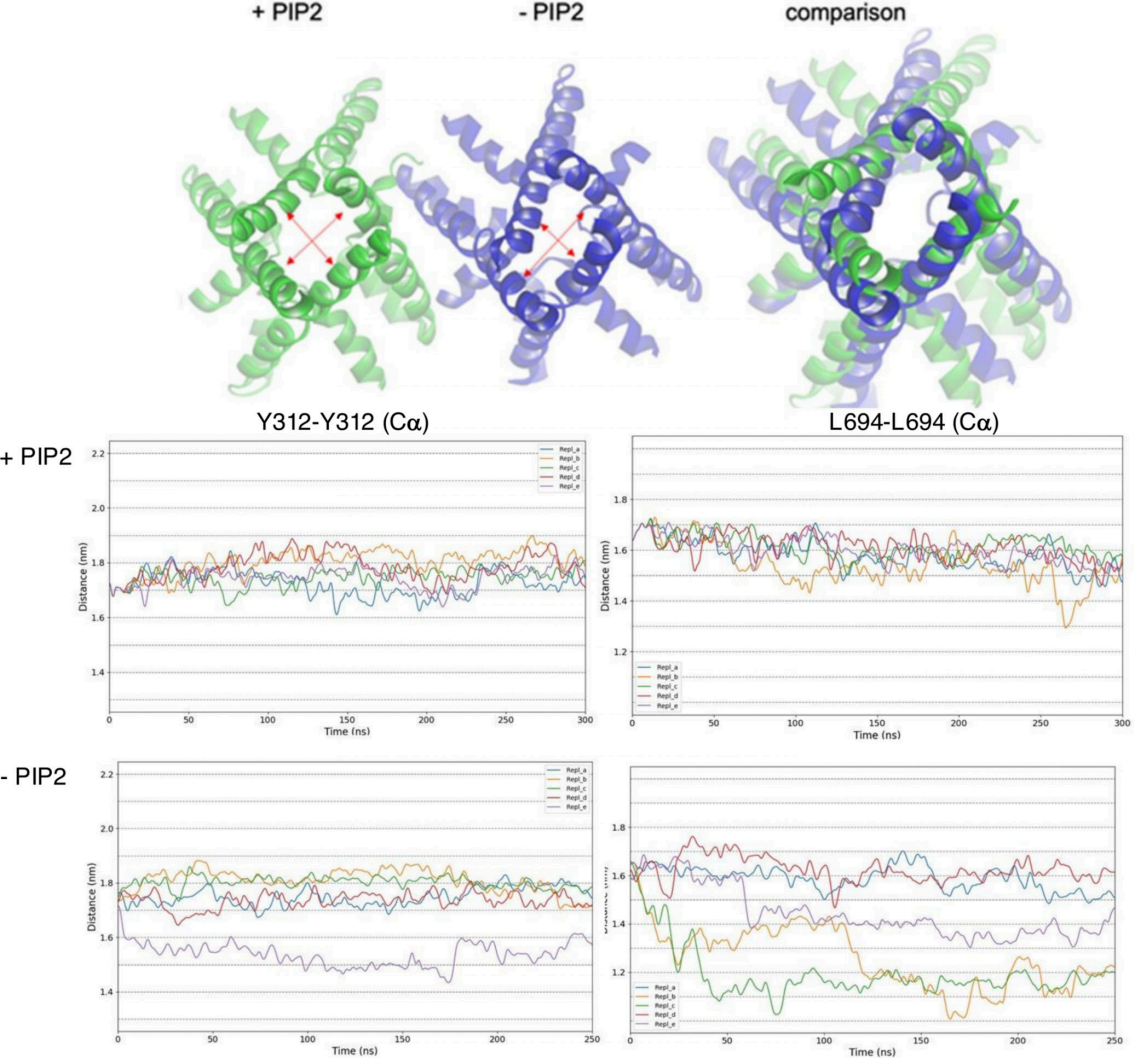
The symmetry change at the hydrophobic gate, induced by PIP2 unbinding.

Fig. S1 shows Na^+^ ion permeation traces for simulations in LysM, demonstrating that ion permeation exhibits no significant lipid dependence in the simulations. Fig. S2 shows the development of the super-open state of the hTPC2 SF with SF-diameter-over-time plots and probability distribution histograms compared to the cryo-EM state. Fig. S3 shows the rate of permeation events versus SF diameter at a voltage of −350 mV. Fig. S4 shows the symmetry change at the hydrophobic gate, induced by PIP2 unbinding. Table S1 provides information about the LysM composition. Table S2 contains a summary of all the simulations performed in this study including length of the simulations, ion concentrations, voltage, and permeation events. Video 1 and Video 2 show the conformational change between PIP2-bound and apo-hTPC2 on PCA modes 1 and 2. Video 3 shows frequent transitions of the ions between the individual hydrophilic-cluster–forming residues (N305, N687, T308) within the cavity, while remaining bound to the cluster overall, as well as several knock-on events. Video 4 shows the collapse of the external loops of hTPC2 in the absence of the disulfate bond between C623.

## Results

### Ligand-dependent opening of the channel at both the selectivity filter and the hydrophobic gate

To investigate the impact of PIP2 binding on the conformation of hTPC2, we first performed simulations of full-length hTPC2 in a POPC bilayer with and without bound PIP2. Both sets of simulations were initiated from the PIP2-bound cryo-EM structure of hTPC2 (PDB ID: 6NQ0), referred to as open-state structure in the PDB, with PIP2 bound to its binding pocket or removed, respectively. In the PIP2-bound simulations, we observed a substantial further dilation at the luminal SF region of hTPC2, increasing the dynamic pore diameter of the cryo-EM structure from ∼4 to 6 Å to ∼12–14 Å (Fig. 2), leading to a highly conductive form of hTPC2 under membrane voltage. The key factor behind this widening was a rearrangement of both, the twin conserved asparagine residues N653 and N654 from filter II and the twin conserved asparagine residues N273 and N274 from filter I (Fig. 2, A and B). Whereas the side chains of N653 and N654 point into the pore entrance in the cryo-EM structure, they reorientated tangentially to the pore in the simulations. Especially the side chains of N653 and N274 formed tight inter-subunit H-bonding interactions, dilating the luminal SF entrance (Fig. 2, C and D). The super-open state was subsequently sustained in all of our initial simulations with bound PIP2 that showed the asparagine rearrangement at the SF entrance (four out of five simulations in the initial set, see Fig. S2). In the simulations without bound PIP2, by contrast, no such transition was observed at the SF (five out of five simulations). Moreover, when repeated under different voltage ranges, unliganded hTPC2 consistently showed no permeability for ions. Accordingly, dilated pore diameters including the super-open SF state support an increased permeation rate across hTPC2 under voltage (Fig. S3).

Comparing our simulations of apo- and liganded TPC2 further, we found that, in most of the simulations, the apo form of TPC2 also adopted a more closed state at the cytoplasmic hydrophobic gate, which is located at the bundle crossing of the channel (Fig. S4). Many of the apo hTPC2 states showed a transition from an open, pseudo-C4 symmetric conformation toward a C2 symmetric form, in which the minimum internal diameter of the hydrophobic gate (including side chains) was markedly reduced from 4 to 0 Å.

### Coupling between PIP2 binding and channel opening

To examine the coupling between ligand binding and pore dilation, we investigated correlated motions in simulations of full-length apo-hTPC2 and the liganded open states, all initiated from the conformation captured by the cryo-EM structure. The initial set of simulations contained five replicates with and five replicates without the ligand. Among the ligand-bound structures, the channel adopted the super-open state of the SF in four out of five replicate simulations, together with a high conductance under voltage.

**Figure 3.**
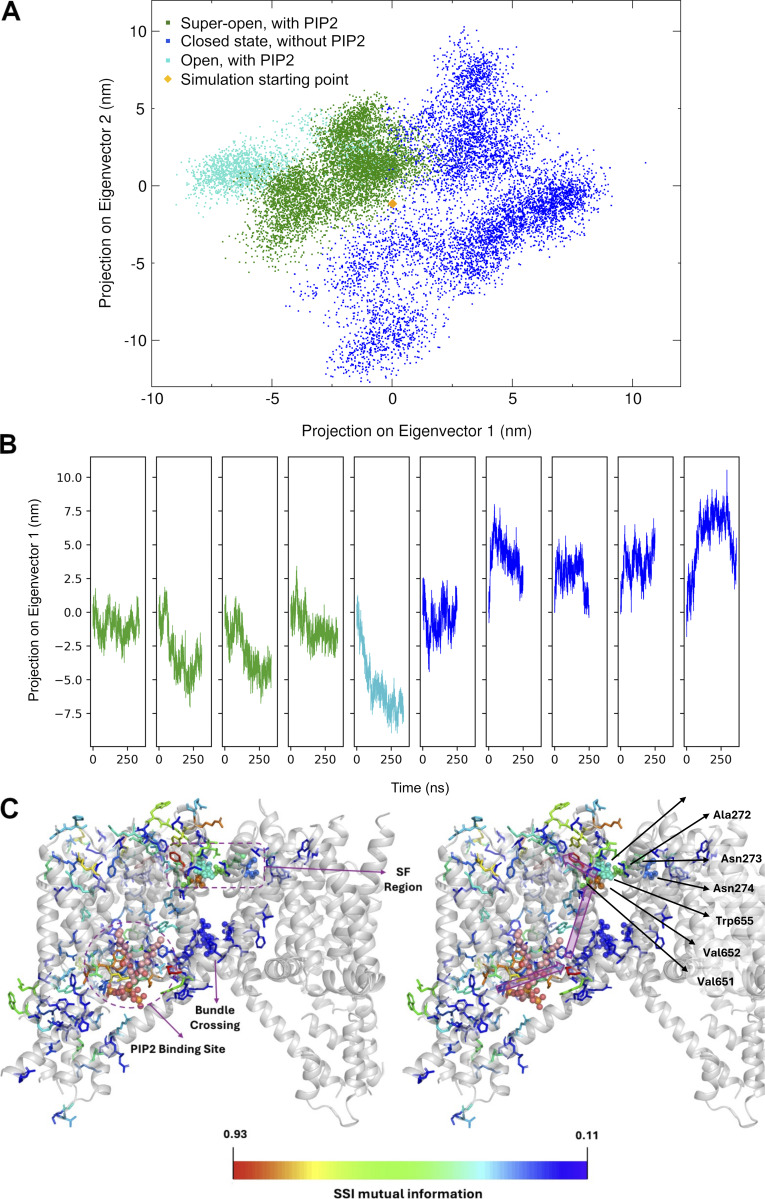
**Principal component and mutual information analysis of global conformational changes induced by PIP2 unbinding. (A)** Projection of the hTPC2 conformations onto the first two eigenvectors of a PCA of all trajectories in the set, showing a separation of the states in this subspace (super-open PIP2-bound state, green; apo-closed state, blue; open state as in the cryo-EM structure [non-conductive], cyan). **(B)** Time evolution of the projection on eigenvector 1 for all trajectories; color code as in A. **(C)** State-specific mutual information (SSI) analysis of the allosteric coupling between the PIP2 binding site, the bundle crossing, and the SF (color spectrum from blue, 0.11 bits, to red, 0.93 bits of mutual information). The location of the residues coupled with the ligand status indicates the formation of allosteric pathways between the ligand binding site, the bundle crossing, and the SF (magenta arrows, right). Key residues in the SF are labeled; important conformational switches in the SF, the bundle crossing region, as well as the ligand are shown in ball-and-stick representation. The values are symmetric between the subunits; only one subunit is shown in the color code for clarity.

To investigate the major correlated protein motions contributing to the opening/closing transition of the channel, we performed a PCA of the full set of these trajectories. Fig. 3 A shows a projection of the trajectories on eigenvectors 1 and 2 obtained from the PCA. The first two PCA eigenvectors were linked to eigenvalues of very similar magnitude (14.74 versus 13.12 nm^2^), together explaining about 28% of the total variance across the trajectories. The first 10 eigenvectors covered about 64% of the variance, showing that further modes may contribute to the overall transition.

The PIP2-bound states, competent to conduct ions, occupied a different region within the space spanned by eigenvectors 1 and 2, as compared with the unliganded states. In general, the trajectories in the liganded states were more confined in the projection and tended toward slightly lower values on eigenvector 1 (see also Fig. 3 B), whereas the replicate simulation that did not adopt a super-open state slightly separated from the remaining liganded replicates.

Analyzing the correlated protein motion on eigenmodes 1 and 2, we found that these modes mainly represented the opening and closing of the hydrophobic gate and the associated symmetry transition from pseudo-C4 toward C2 symmetry in the absence of PIP2. This motion of the pore-forming helical bundle was linked to a rotation of the VSDs, which contain the PIP2 binding sites, toward each other, and to a conformational change of the SF backbone. The PIP2-binding VSD of each subunit rotated toward the non-PIP2 binding VSD, again moving in a direction from pseudo-C4 toward the C2 symmetry of the overall apo-protein structure (Video 1). During this transition, an expansion of the space between helices S4, S4–S5, and S6, which houses the PIP2 binding site, was observed in the apo-state.

**Video 1.** **The conformational change between PIP2-bound and apo-hTPC2 on PCA modes 1 and 2.** Playback speed is 10 fps.

To investigate further the allosteric pathway linking the PIP2 binding site, the hydrophobic gate, and the SF, we used a mutual-information–based analysis (SSI) to determine backbone and side chain conformational changes across hTPC2 that correlated specifically with the presence or absence of the PIP2 ligand (She et al., 2019; Wang et al., 2012). SSI calculates the mutual information between an external variable (here the ligand status) and the distribution of conformational states of each residue. Fig. 3 C shows the average mutual information shared between the ligand status and each side chain and backbone conformation in the two hTPC2 subunits as color code, in which SSI values above average are depicted in a color spectrum from blue to red (maximum). From the PIP2 binding pocket, residues that couple to the ligand status align along several pathways connecting the binding pocket to both the SF and the bundle crossing (Fig. 3 C, right). These routes can be interpreted as forming allosteric pathways that link PIP2 binding with the channel opening mechanism.

As expected, the specific conformational state of the PIP2 binding region exhibited a high correlation with the presence or absence of the ligand, especially affecting the side chains of this area. The SSI analysis highlighted an array of highly coupled residues along helix II-S6 which, via a short stretch of helix I-S5, linked the PIP2 binding site with the pore helix and SF. The SF itself, especially its backbone, thus showed a strong conformational response to the ligand status of hTPC2 (SF residues shown as spheres). We interpret this alignment of strongly coupled residues as an allosteric pathway connecting the ligand pocket with the SF. The coupling between the PIP2 binding site and the SF involved key conformational switches at residues S681 and W685 in helix II-S6, and M650 and L649 in the II-P1-P2 domain.

Furthermore, several pathways of individually slightly lower mutual information, but acting in parallel, coupled the hydrophobic gate on helices S6 and S5 (I and II) with the ligand binding region, with residues K203 and L206 in the I-S4-S5 linker, F315 in I-S6, M579 and L575 in the II-S4-S5 linker, F582 in II-S5, and L692 and I693 in II-S6 forming parallel bridges of conformational switches toward T308 and L694 (shown as spheres) at the bundle crossing (hydrophobic gate) (Gan and Jiang, 2022). Overall, our SSI analysis therefore suggests the existence of specific allosteric pathways between the ligand binding pocket, the hydrophobic gate, and the pore helix/SF in hTPC2.

### Ion conduction through the inner hTPC2 pore

**Figure 4.**
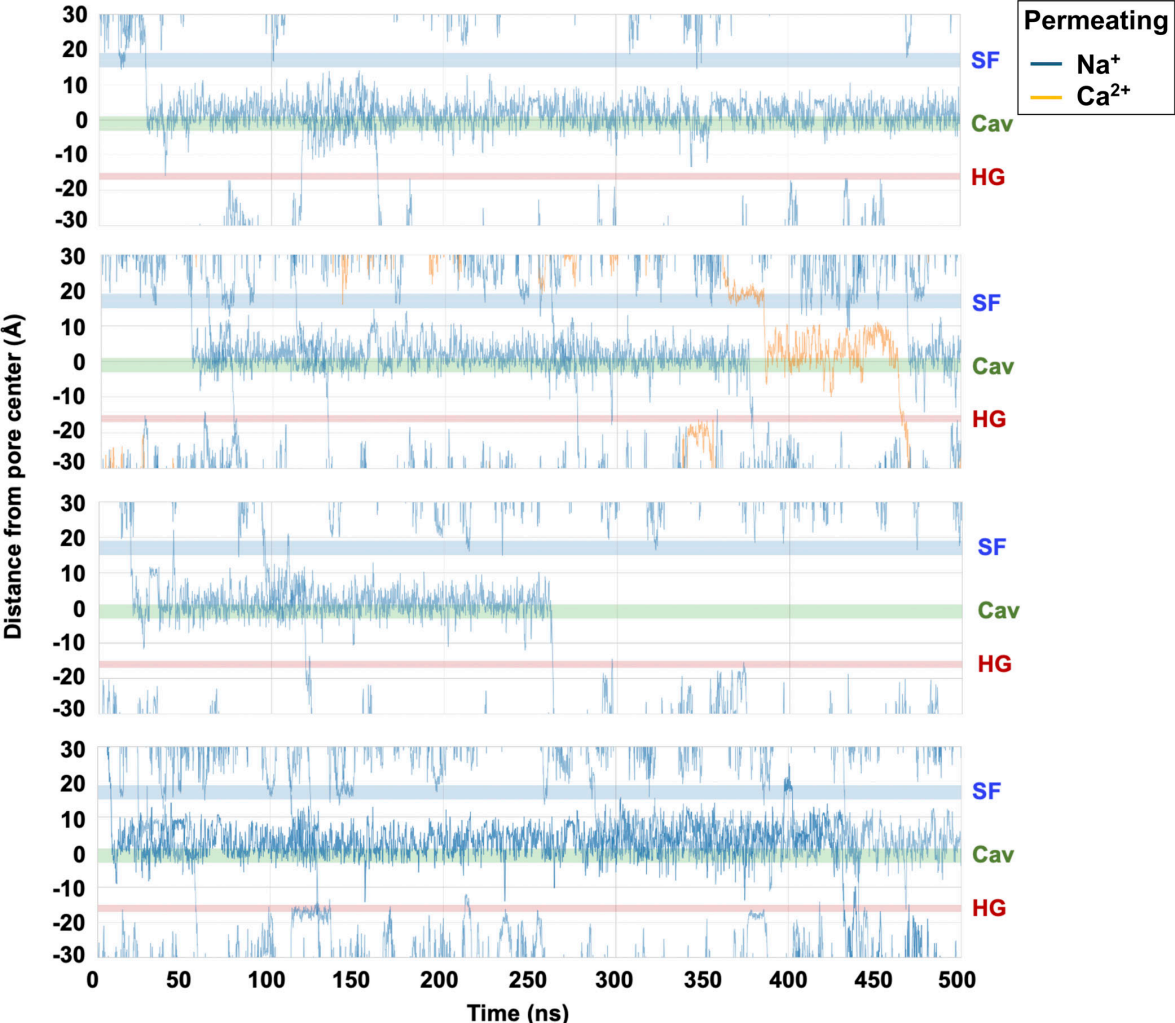
**Permeation traces of 14 Na** ^**+**^ **ions (blue) and one Ca** ^**2+**^ **ion (orange) across the pore of hTPC2 at a voltage of −70 mV.** Locations marked in the channel are SF, Cav (cavity center), and HG (hydrophobic gate). One out of five replicates did not show any permeating ions on this time scale.

We next simulated the open state of hTPC2 under a range of ionic conditions and various transmembrane voltages (V_m_; for all simulation conditions, see Table S2). In fivefold repeated 500-ns simulations at the lowest applied V_m_ of −70 mV in mixed equimolar NaCl/CaCl_2_ solutions (0.3/0.3 M), we observed the complete permeation of 14 Na^+^ ions versus only one Ca^2+^ ion across hTPC2 (Fig. 4 shows the permeation traces of the ions traversing the pore; in one replicate simulation we did not observe any permeation). As can be seen, the major interaction site between hTPC2 and the Na^+^ ions is the central channel cavity. Contrary to many other cation channels, the luminal SF only showed relatively short-lived interactions with the ions, by contrast.

**Figure 5.**
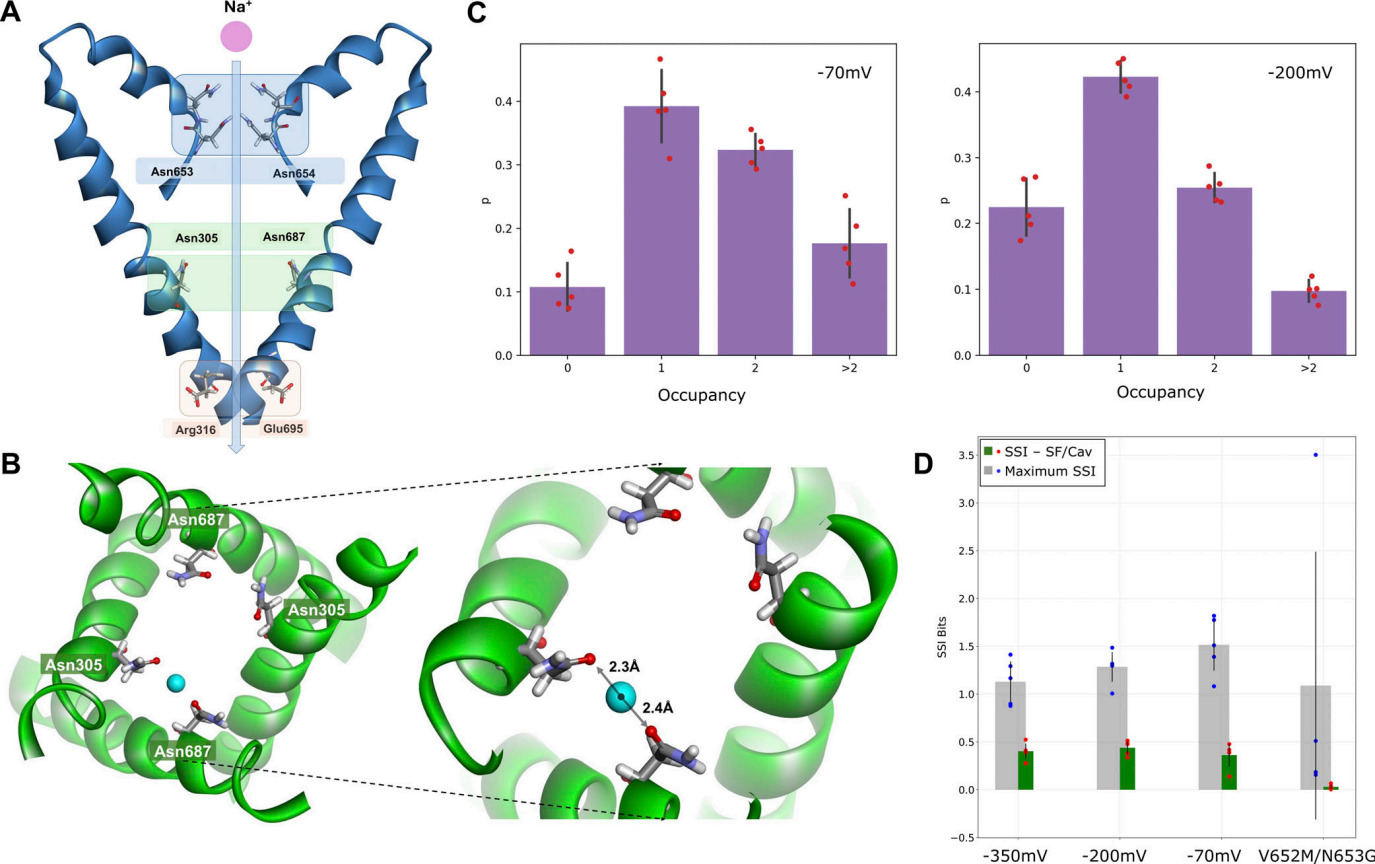
**(A)** Key interaction sites for Na^+^ ions within the hTPC2 pore are the SF (blue shade, residues N653 and N654 from filter-II are highlighted), the central cavity (green shade, residue N687 from 6-TM-2 is highlighted; N305 is located in the same pore region on 6-TM-1), and the hydrophobic gate region (red shade; shown are the externally facing residues E695 from 6-TM-2 near the gate for clarity; R316 is located in the same region on 6-TM-1). **(B)** Close-up view of residues N305 and N687 from two subunits, a Na^+^ ion binds between the asparagine side chains from one subunit but can change to other hydrophilic side chains in the cavity hydrophilic cluster; additional ions can occupy these binding sites. **(C)** The cavity is frequently occupied by more than one Na^+^ ion at lower voltage regimes (purple bars show the mean occupancy from five repeat simulations ± SD at a voltage of −70 and −200 mV, respectively). The individual simulation results are shown in red. **(D)** Mutual information between the time when changes in SF and cavity binding site occupancy occur (the green bars show the average SSI from five repeat simulations ± SD at voltages of −350, −200, and −70 mV; individual simulation results in red). Substantial mutual information is seen in all voltage ranges for the hTPC2 wild type (the gray bars denote the maximum theoretical mutual information attainable ± SD, individual data points in blue). This finding signifies a considerable contribution of a distant knock-on mechanism to conduction efficiency. By contrast, in the SF mutant V652M/N653G, the knock-on mechanism is disrupted, likely by abolishing a distinct SF binding site, leading to a decreased Na^+^ conductance.

The importance of the central cavity for ion conduction in hTPC2 has been recognized previously (Milenkovic et al., 2021); however, rather than forming a non-interacting aqueous volume occupied with a single ion, we found that a cluster of hydrophilic residues in the middle of the cavity (location marked in green in Fig. 5 A) acted as a distinct binding region for Na^+^ ions. This cluster is composed of the conserved asparagine residues N305 and N687 at opposite sides of the cavity and the threonine residue T308 from each subunit (Fig. 5 B). Additionally, we recorded interactions between the ions and the backbone oxygen atoms of V651 and V652 at the cavity-facing end of the SF.

In our simulations, the cations interacted closely with the hydrophilic cluster over substantial time spans (Fig. 4, marked as Cav). Of note, the ions showed frequent transitions between the individual cluster-forming residues within the cavity while remaining bound to the cluster as a whole (Video 2). Conversely, the cations did not display any major interactions with the protein matrix during their traversal of the cytoplasmic hydrophobic gate region (Fig. 4, HG). A summary of all recorded ion permeation events in our simulations is shown in Table S2.

**Video 2.** **The conformational change between PIP2-bound and apo-hTPC2 on PCA modes 1 and 2.** Playback speed is 10 fps.

The existence of multiple cation binding sites within the central cavity also led to the phenomenon that several ions were often observed to occupy the central cavity at the same time, especially at lower voltages. At −70 mV, for instance, the probability of two or more ions to be bound within the cavity was about 50% (Fig. 5 C).

Both at −70 and −200 mV, Na^+^ ions entering the cavity from the SF were often seen to permeate rapidly when the cavity was already occupied with one or more ions and its binding sites were therefore saturated (see, e.g., permeation traces in Fig. 4, bottom panel). This “overflow” process of the cavity binding capacity allowed for an enhancement of the Na^+^ conduction rates despite the relatively long average residence times of cavity-bound Na^+^ ions. An observation of single ions bypassing an ion occupying the cavity was previously made by Milenkovic et al. where two fast modes of conduction were described—a bypassing mechanism and a distant knock-on mechanism (Milenkovic et al., 2020), while a slow mode involved only a single ion. In our simulations, by contrast, the cavity showed a higher level of occupancy in general, and a degree of cooperativity between ion occupancy changes in the SF and the cavity (e.g., correlated unbinding at both binding sites) existed under all voltage regimes: The excess mutual information between binding site changes amounted to ∼0.4 bits for Na^+^ conduction, while the maximum possible level of cooperativity was calculated to be between 1.1 and 1.5 bits (Fig. 5 D). This indicated a relatively constant contribution of a distant knock-on mechanism to the efficiency of ion permeation in hTPC2 under all voltage regimes we investigated; however, the overflow mechanism of a highly occupied cavity appeared to play the key role within the most physiologically relevant voltage range.

### Luminal approach pathway into the SF and impact of pH on ion conduction

**Figure 6.**
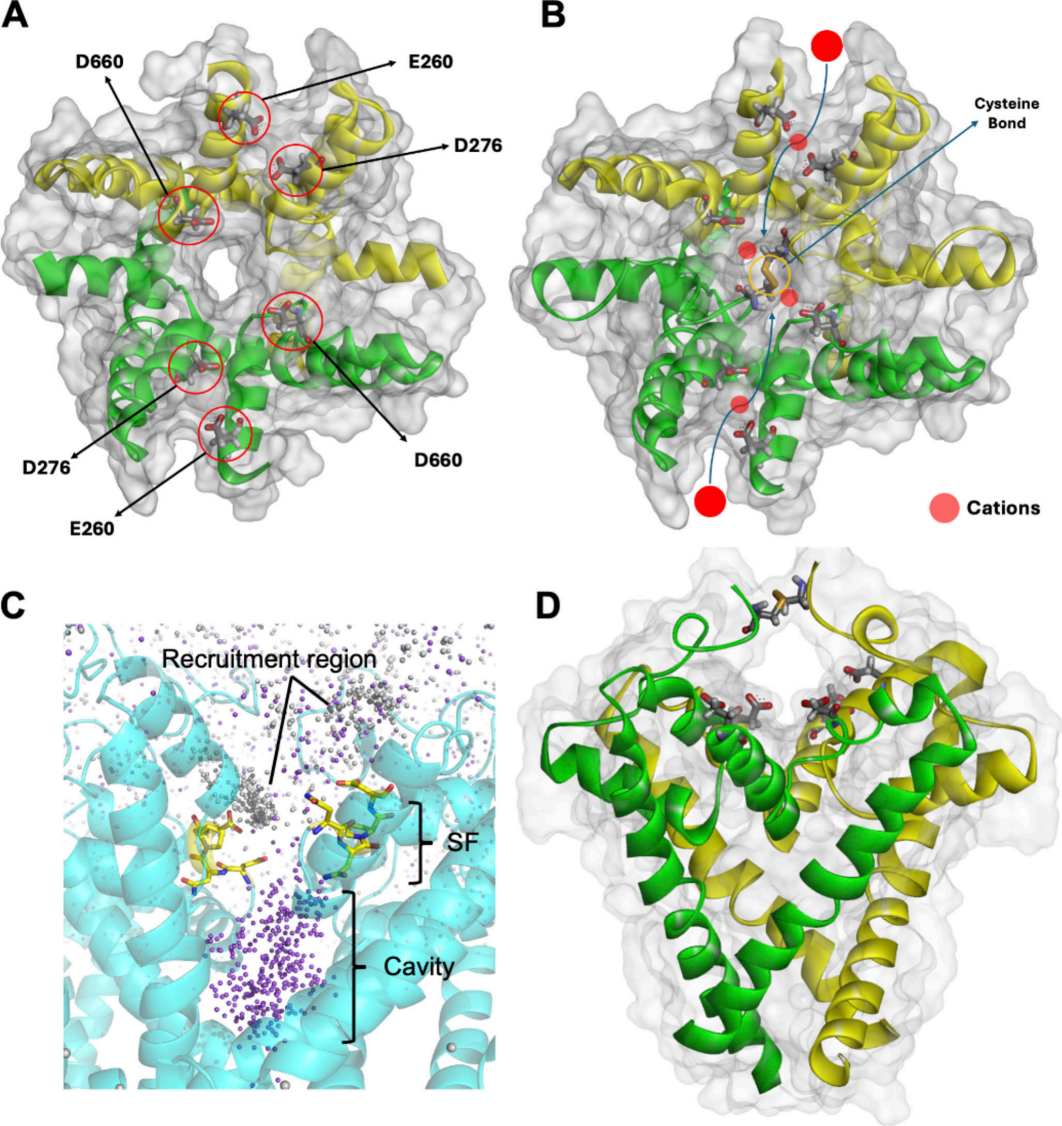
**Entry pathways for cations entering the hTPC2 SF. (A)** Top view of the luminal entrance highlighting the acidic residues in each subunit that recruit cations. **(B)** Horizontal approach pathway for cations toward the SF; the C623/C623 disulfide bond is shown. **(C)** An overlay of 200 frames from a simulation at −70 mV shows the accumulation of Na^+^ (purple) and Ca^2+^ ions (gray) near residues D660 at the top of the SF; the channel cavity is occupied only by Na^+^ ions. **(D)** Location of the C623/C623 disulfide bond forming a “roof” above the SF entrance.

Within the second domain (II) of the hTPC2 homodimers, the S5 helices and the pore-forming helices P1–P2 are connected by two lumen-facing external loops (Fig. 1 D). These two loops consist of 37–38 residues and are interlinked via a disulfide bond between the C623 residues of each subunit, which are specific to hTPC2. Similar cysteine residues are absent from the corresponding regions in mTPC1 and atTPC1. Unlike in other ion channels, the permeating ions therefore cannot approach the hTPC2 SF from the luminal side along an extension of the pore axis, as the disulfide bond and the loops block this entryway. In our simulations, the ions approached the SF laterally on two pathways formed between the P1 and P2 helices of the subunits. These pathways are lined by negatively charged aspartate (D660, D276) and glutamate (E260) residues, which act as attractive sites for cations (Fig. 6, A and B).

In simulations in which the disulfide bond was removed (by a C623A mutation), the loops folded back inward and closed the pore from the luminal side in the mutant. Furthermore, P622 blocked the pore entrance and presented a physical barrier for the incoming ions (Video 3). The C623 disulfide bond therefore appears important for maintaining the luminal channel entrance in a conformation accessible to ions (Video 4).

**Video 3.** **Frequent transitions of the ions between the individual hydrophilic-cluster forming residues (N305, N687, T308) within the cavity, while remaining bound to the cluster overall, as well as several knock-on events.** Playback speed is 10 fps.

**Video 4.** **The collapse of the external loops of hTPC2 in the absence of the disulfate bond between C623.** Playback speed is 10 fps.

As hTPC2 resides in the endolysosomal membrane, its luminal side is exposed to a highly acidic environment, whereas its cytosolic side faces a neutral pH of 7.4. The drastic local pH environment on the luminal side may affect its function and mechanism of ion conduction. To investigate the effect of low pH on the channel, we simulated near the lower pH limit of the lysosomal lumen, focusing on highly acidic (pH 3.5) and acidic (pH 4.5) states on the luminal side (see Table S2). For each condition, we conducted fivefold replicated simulations under a voltage of −350 mV in equimolar mixed solutions of NaCl and CaCl_2_ (0.3/0.3 M) over a total time span of 2.5 µs for each condition.

In the highly acidic condition (pH 3.5), all Asp and Glu residues leading to the luminal channel entrance were protonated. In these simulations, we did not observe any ion passage over the total simulation time. This is likely due to the reduced electrostatic attraction toward cations once when these residues are neutral. By contrast, at a higher pH of 4.5, only the Glu residues were protonated, while D660 and D276 remained deprotonated. Under these conditions, we observed 42 Na^+^ and 5 Ca^2+^ to permeate hTPC2 over the full simulation time scale. Our results therefore suggest that the function of hTPC2 is maintained at the lower pH values typical for the endolysosomal lumen, which range from 6.5 to 4.5 (Hu et al., 2015). Selectivity for Na^+^ permeation is maintained, but the permeation ratio is slightly reduced. This result may offer an explanation for the slightly lower permeation ratio measured in whole lysosomal recordings of hTPC2 with P_Na_:P_Ca_ ≈ 10:1 compared with a ratio of P_Na_:P_Ca_ ≈ 17:1 measured in plasma membrane recordings (Shah et al., 2022).

### Mutations at filter II abolish the Na^+^ selectivity of hTPC2

In simulations of hTPC2 in mixed NaCl/CaCl_2_ solutions (0.3/0.3 M) at neutral pH and membrane voltages of −70, −200, and −350, the channels displayed a high selectivity for Na^+^ ions, with an average permeability ratio of P_Na_:P_Ca_ = (17.1 ± 6.8):1 (SEM) across these voltages (total simulated time: 2.5 μs for each voltage). These findings are in excellent agreement with the experimentally observed selectivity for PIP_2_-bound hTPC2 of P_Na_:P_Ca_ ≈ 17:1 in plasma membrane recordings and P_Na_:P_Ca_ ≈ 10:1 in whole lysosomal recordings (Shah et al., 2022).

**Figure 7.**
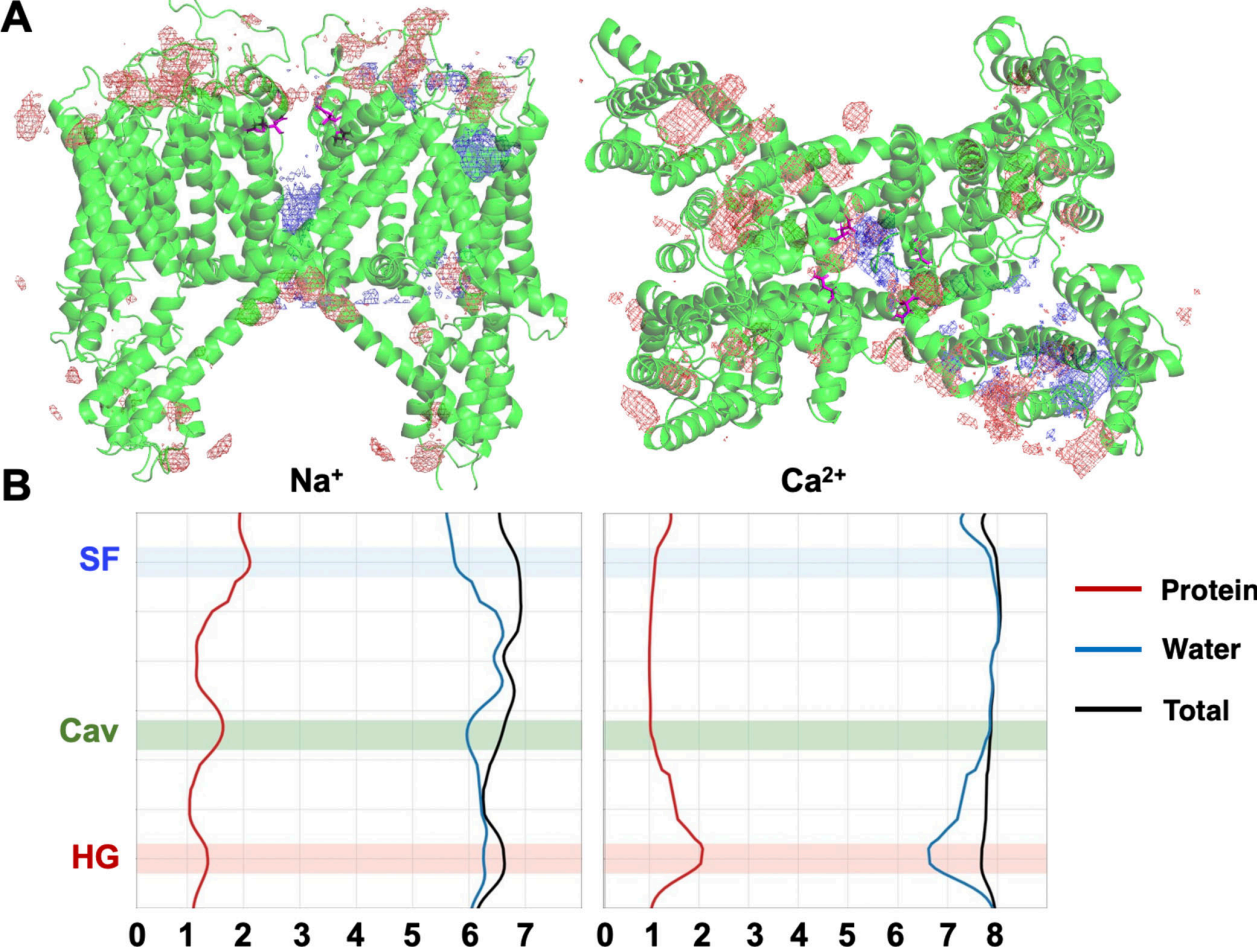
**(A)** Density of Ca^2+^ ions (red mesh) and Na^+^ ions (blue mesh) around the hTPC2 protein matrix from simulations performed in equimolar dicationic solution. Left: side view; right: view from the lumen. **(B)** Interactions of Na^+^ and Ca^2+^ ions with water oxygen atoms (blue) and protein oxygen atoms (red) within their first solvation shell. The main interaction sites with hTPC2 at the SF, central cavity (Cav), and hydrophobic gate (HG) are marked. Na^+^ ions show closer interactions with the protein, especially at the SF and in the central cavity, and a higher degree of desolvation.

Since neither cation type exhibited long-lived interactions with the channel’s SF upon their traversal, our observations raised the question of how this selectivity is achieved, and therefore, we examined the favored binding regions for Na^+^ and Ca^2+^ ions in the mixed solutions. As shown in Fig. 7 A, the ion density maxima found for Na^+^ ions near the protein matrix are located on the pore axis, within the central cavity, and throughout the VSD. In contrast, on the luminal side of the channel, the main Ca^2+^ binding sites occur off the pore axis near acidic clusters consisting of residues D276, D660, and E260. The Ca^2+^ density maxima are not observed near the channel entrance on the pore axis, the SF, or the central cavity, such that the local Ca^2+^ concentration is depleted compared with the local Na^+^ concentration at the luminal entrance to hTPC2. Like Na^+^ ions, all Ca^2+^ ions that entered the cavity first bound to the hydrophilic cluster region at N305, N687, and T308, and subsequently fully permeated the channel, driven by V_m_. In our simulations, we observed that permeating cations interacted more closely with filter II than with filter I. As shown by the solvation profile of both ion types (Fig. 7 B), the SF matrix replaced one water molecule in the hydration shell of Na^+^ through transient interactions, especially with the asparagine side chains of filter II (N653 and N654). In contrast, these interactions are absent in the case of Ca^2+^ ions, which permeated the SF in a fully hydrated state.

**Figure 8.**
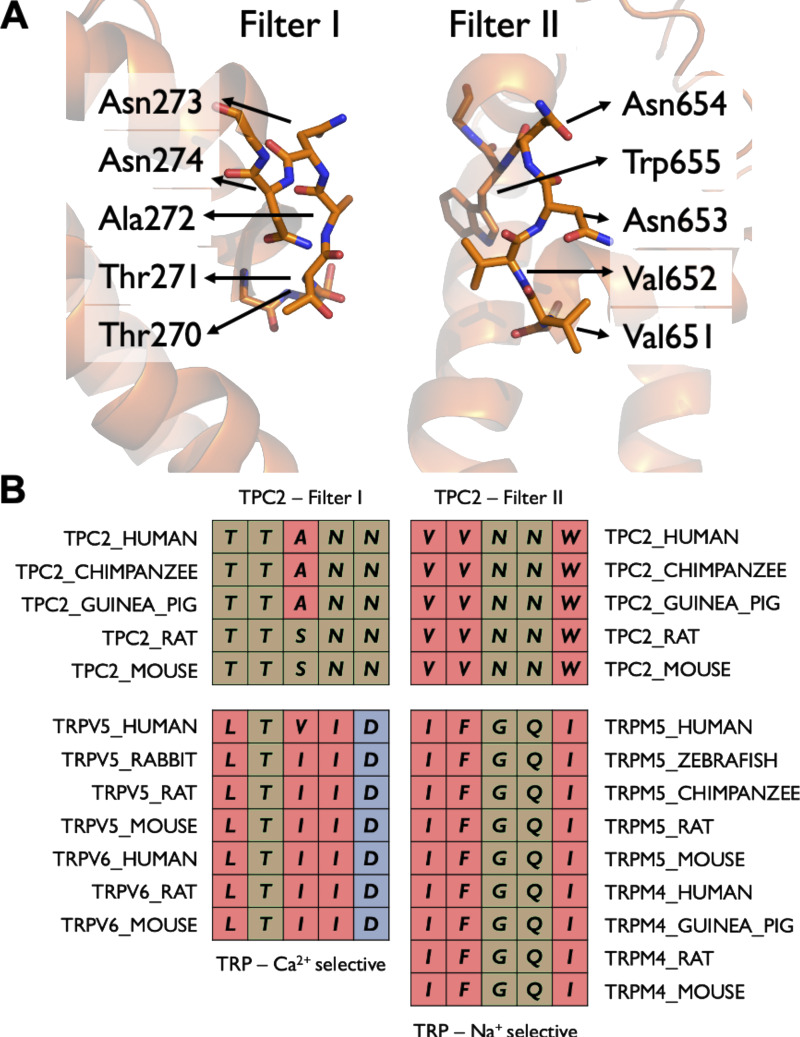
**The SF of hTPC2 displays similarities to both Ca^2+^-selective and Na^+^-selective channels. (A)** Close-up view of adjacent filter I and filter II structures forming the SF. **(B)** Sequence comparison between filter I and filter II and Ca^2+^-selective or Na^+^-selective TRP channel SF sequences, respectively. Residue color code: Hydrophobic, red; polar and glycine, tan; acidic, blue.

In addition to the lowered local concentration at the SF, Ca^2+^ ions were typically less able to pass through the SF as well as the transition region between the SF and the cavity. The sequence of both hTPC2 filters, I and II, contains a pair of neutral asparagine residues instead of negatively charged aspartate residues. Aspartate residues are often related to tight Ca^2+^ interactions and are present, for example, in the SFs of the Ca^2+^ selective channels TRPV5 and TRPV6 (Fig. 8, A and B). By contrast, the sequence of filter II resembles the SF sequences of TRPM4 and TRPM5 channels, which are both highly selective for monovalent cations versus Ca^2+^ (Owsianik et al., 2006; Ives et al., 2024).

The plant channel atTPC1 shows a slight preference for conducting Ca^2+^ over Na^+^ (permeability ratio: ∼5:1) (Hedrich et al., 2023; Hedrich and Marten, 2011). In Filter II of atTPC1, the positions corresponding to N653 and V652 in hTPC2 are occupied by glycine and a methionine residue, respectively, and thus we simulated a hTPC2 double mutant N653G/V652M.

In fivefold repeated 500-ns simulations at a voltage of −350 mV, we observed the selectivity of the mutant channel to be nearly abolished. Overall, we found that the permeation rate for Ca^2+^ was largely unaffected by the mutation (seven permeation events in total versus nine in WT), whereas the permeation rate for Na^+^ was substantially reduced (16 permeation events versus 70 in WT). This result suggested a key role for N653 in facilitating Na^+^ conduction and, as a consequence, Na^+^ selectivity. Indeed, we found that the absence of an SF binding site for Na^+^ resulted in the disruption of knock-on cooperativity between the SF and the cavity (Fig. 5 D). This, in turn, reduced the conduction rate of Na^+^, while no major changes were seen for Ca^2+^ permeability. Furthermore, the transition zone between the SF and central cavity is formed by the hydrophobic residue pair V651 and V652 in filter II, whereas a pair of polar threonine residues, T270 and T271, line this region in filter I. Our previous simulations of the TRPM5 channel suggested that traversing such a hydrophobic funnel region incurs a lower energy penalty for monovalent cations than for divalent cations, likely due to the difference in Born energy (Ives et al., 2024; Born, 1920). In TRPM5, mutating F904 in the transition zone between SF and cavity greatly reduced the channel’s selectivity for monovalent cations. We therefore studied the effect of mutating the transition zone of hTPC2 filter II.

In a V651T/V652T double mutant, simulated under a voltage of −350 mV in equimolar NaCl/CaCl_2_ solutions (0.3/0.3 M), we observed the Na^+^ selectivity of hTPC2 to be fully abolished. During a total simulated time of 2.5 μs, 15 Na^+^ ions and 17 Ca^2+^ ions permeated the channel. Compared with the other double mutant investigated, N653G/V652M, the lower permeation rate for Na^+^ with respect to the WT channel was maintained in V651T/V652T, but the permeation rate for Ca^2+^ was approximately doubled.

## Discussion

TPCs are cation-selective ion channels that enable the permeation of ions from the luminal side of endolysosomes to the cytoplasm (Wang et al., 2012). They play crucial roles both physiologically and as drug targets (Jaślan et al., 2023b). TPCs are known for their ability to alter their ion selectivity dependent on the type of ligand bound, which is a unique phenomenon for ion channels (Jaślan et al., 2023a; Wang et al., 2012). Unlike most other Na^+^ and Ca^2+^ channels, TPCs do not contain negatively charged residues in their SF, and the filter II transition zone between the SF and the central cavity forms an unusually hydrophobic structure (Fig. 8, A and B). Due to the dimeric nature of TPCs, the SF is composed of a pair of two distinct SF sequences, filter I and filter II. Through extended *in silico* electrophysiology simulations, we studied the permeation of Na^+^ and Ca^2+^ ions across WT hTPC2 bound to the gating ligand PIP2 as well as SF variants which alter the channel’s ion selectivity. In our simulations, all of the permeating ions interacted chiefly with filter II while traversing the SF and subsequently occupied the central cavity for substantial amounts of time.

Whereas the WT exhibited Na^+^ selectivity with a Na^+^/Ca^2+^ permeation ratio compatible with prior experiments (Shah et al., 2022), mutations that either transformed the SF-cavity transition zone into a hydrophilic region (V651T/V652T mutant) or altered the main interaction site between the SF and permeating ions (N653G/V652M mutant, yielding an atTPC1 filter sequence) displayed a substantially diminished selectivity for Na^+^. In the case of the V651T/V652T mutant, Na^+^ selectivity was completely abolished. We, therefore, conclude that the sequence and structure of filter II and its transition zone to the central cavity is the major element governing Na^+^-selectivity in hTPC2. It should be noted, however, that we determined the permeability ratio of cations in the simulations by the number count of individual permeation events in symmetric di-cationic solutions, whereas experimentally, it has been derived from the reversal potential in asymmetric dicationic solutions using a Goldman–Hodgkin–Katz-type equation (Hille, 2001; Wang et al., 2012).

The V651T/V652T mutation of filter II, which emulates important elements of the sequence of filter I, led to an increase in Ca^2+^ permeation, such that our simulations suggest that closer interactions between the ions and filter I may be responsible for the increased Ca^2+^ flow in NAADP-bound hTPC2 (Gunaratne et al., 2023; Pitt et al., 2016). However, we observed all cations in our simulations to interact more strongly with filter II. We, therefore, speculate that a change from Na^+^ to Ca^2+^ selectivity could be furnished by a rearrangement of the SF into a geometry that reduces the impact of filter II and enhances the interaction of ions with filter I. Of course, further research will be needed to establish the conformational basis for such a potential SF form.

Comparing TPC2 filters I and II with the evolutionarily related Ca^2+^-selective and monovalent-cation selective TRP channels (Fig. 8 B), it becomes apparent that filter II shares important sequence and physicochemical features with the SF of the monovalent-selective TRPM4 and TRPM5 channels (Ives et al., 2024). The similarity level agrees with our observations that the major interacting site of hTPC2 with Na^+^ ions in the SF is formed by filter II and that the WT shows a high degree of Na^+^-selectivity in most voltage ranges in our simulations.

By contrast, the sequence of filter I seems to be unique and only shares a limited number of features with the SFs of the two Ca^2+^-selective TRP channels TRPV5 and TRPV6. In particular, filter I is more hydrophilic overall than the SFs of TRPV5 or TRPV6, and negatively charged residues are absent. Mutations of filter II that bring its sequence closer to that of filter I lead to the abolishment of Na^+^-selectivity, such that it can be concluded that the filter I sequence likely explains the Ca^2+^-conductance of hTPC2.

The selectivity for Na^+^ in the WT, as compared to the investigated mutants, is further enhanced by multiple factors that enhance Na^+^ permeation rates. First, a distant knock-on mechanism between Na^+^-favoring interaction sites in the SF and cavity is in operation, as demonstrated by our mutual information analysis, which increases permeation efficiency. Mutation of the main Na^+^ binding site in the SF removes the cooperativity between the two binding sites and leads to a greatly reduced permeation rate for Na^+^. Second, especially at voltages representative of the physiological environment of hTPC2, the cavity can house multiple ions, frequently more than two at −70 mV. When the binding sites in the cavity are fully occupied, single ions can rapidly traverse the cavity on their own without being slowed down by further interactions. Additionally, the stochastic movement of multiple ions within the cavity leads to situations where the distance between the ions is reduced, and the repulsion between the close charges further facilitates exit by a cavity “overflow” mechanism.

A third factor playing a role in Na^+^-selectivity is the location of the main interaction sites for Ca^2+^, which are situated away from the main pore axis on the luminal side, whereas the major Na^+^ binding sites are on filter II and in the cavity. A chain of negatively charged residues (E260, D276, and D660) attracts both cation types toward the pore on the luminal side in a perpendicular direction, but mainly Na^+^ ions enter the pore from these locations. Our simulations further show that these acidic residues remain functional in the low-pH conditions typical for the endolysosomal lumen.

It is important to note the dependence of calculated conduction rates and ion selectivity values on the force field used. The binding affinity of ions to biomolecules can show a substantial variation even upon small differences in the Lennard–Jones parameters representing the interaction between the ions and the protein matrix, especially for carboxylate groups (Venable et al., 2013). This could affect the interaction between divalent ions and the protein, in particular (Yoo et al., 2016). Although the multisite model developed by Zhang et al. has been carefully parameterized regarding ion-protein affinity (Zhang et al., 2020), the force fields normally used in MD simulations of ion channels, for example, do not account for electronic polarization effects. One recent approach to approximate polarization effects, published while this work was under review, is the Electronic Continuum Correction method, a force field modification that scales down formal atomic charges, to introduce the polarization of ions in a mean-field way (Hui et al., 2024, *Preprint*). We will address improved ways to include electronic polarization in the ionic models in future work.

Nearly all permeation events we observed required a conformational change at the SF, dilating it substantially further with respect to the cryo-EM structure of hTPC2. This rearrangement was based on the rotation of the N653 and N274 side chains into a pore-tangential orientation, facilitated by H-bonds (Fig. 2). A recent paper, published while the present work was under revision, confirmed our observation of the dilated SF state and its importance for ion conduction (Zaki et al., 2024). The dilated conformation and the key functional role of the central cavity binding site residues were previously reported in our preprint (Şahin and Zachariae, 2023, *Preprint*).

## Supplementary Material

Table S1 provides information about the LysM composition.

Table S2 contains a summary of all the simulations performed in this study including length of the simulations, ion concentrations, voltage, and permeation events.

## Data Availability

The data is available upon reasonable request from the corresponding author.
