# Supplementary material for: In silico characterization of the gating and selectivity mechanism of the human TPC2 cation channel
Source: J Gen Physiol. 2025 Feb 21;157(3):e202313506. doi: 10.1085/jgp.202313506 (PMC11844439; doi:10.1085/jgp.202313506)
Supplement: Table S2 — contains a summary of all the simulations performed in this study including length of the simulations, ion concentrations, voltage, and permeation events. [file jgp_202313506_tables2.docx]

Simulations under various voltage regimes with PIP2 and dicationic solutions (2-fs time steps).

| Dicationic Conditions -700mV with PIP_2_ (2-fs) | | | |
| --- | --- | --- | --- |
|  | Simulation Time | Na^+^ Permeation Events | Ca^2+^ Permeation Events |
| Repl_a | 1μs | 36 | 15 |
| Repl_b | 1μs | 9 | 4 |
| Repl_c | 1μs | 15 | 4 |
| Repl_d | 1μs | 4 | 1 |
| Repl_e | 1μs | 58 | 42 |

| Dicationic Conditions -350mV with PIP_2_ (2-fs) | | | |
| --- | --- | --- | --- |
|  | Simulation Time | Na^+^ Permeation Events | Ca^2+^ Permeation Events |
| Repl_a | 500 ns | 20 | 3 |
| Repl_b | 500 ns | 17 | 2 |
| Repl_c | 500 ns | 14 | 2 |
| Repl_d | 500 ns | 13 | 0 |
| Repl_e | 500 ns | 7 | 3 |

| Dicationic Conditions -200mV with PIP_2_ (2-fs) | | | |
| --- | --- | --- | --- |
|  | Simulation Time | Na^+^ Permeation Events | Ca^2+^ Permeation Events |
| Repl_a | 500 ns | 6 | 0 |
| Repl_b | 500 ns | 6 | 0 |
| Repl_c | 500 ns | 2 | 0 |
| Repl_d | 500 ns | 10 | 1 |
| Repl_e | 500 ns | 6 | 0 |

| Dicationic Conditions -70mV with PIP_2_ (2-fs) | | | |
| --- | --- | --- | --- |
|  | Simulation Time | Na^+^ Permeation Events | Ca^2+^ Permeation Events |
| Repl_a | 500 ns | 4 | 1 |
| Repl_b | 500 ns | 2 | 0 |
| Repl_c | 500 ns | 2 | 0 |
| Repl_d | 500 ns | 0 | 0 |
| Repl_e | 500 ns | 6 | 0 |

| Dicationic Conditions -200mV with PIP_2_ (2-fs) | | | |
| --- | --- | --- | --- |
|  | Simulation Time | Na^+^ Permeation Events | Ca^2+^ Permeation Events |
| Repl_a | 500 ns | 0 | 0 |
| Repl_b | 500 ns | 0 | 0 |
| Repl_c | 500 ns | 0 | 0 |
| Repl_d | 500 ns | 0 | 0 |
| Repl_e | 500 ns | 0 | 0 |

Simulations with different pH conditions (2-fs time steps).

-Only the lysosome-facing residues are protonated/deprotonated accordingly.

| Dicationic Conditions -350mV with PIP_2_  pH 4.5 (2-fs) | | | |
| --- | --- | --- | --- |
|  | Simulation Time | Na^+^ Permeation Events | Ca^2+^ Permeation Events |
| Repl_a | 500 ns | 2 | 0 |
| Repl_b | 500 ns | 13 | 3 |
| Repl_c | 500 ns | 8 | 0 |
| Repl_d | 500 ns | 7 | 0 |
| Repl_e | 500 ns | 12 | 2 |

Simulations with mutations (2-fs time steps)

| Dicationic Conditions -350mV with PIP_2_  N653G/V652M (2-fs) | | | |
| --- | --- | --- | --- |
|  | Simulation Time | Na^+^ Permeation Events | Ca^2+^ Permeation Events |
| Repl_a | 500 ns | 8 | 0 |
| Repl_b | 500 ns | 2 | 3 |
| Repl_c | 500 ns | 0 | 0 |
| Repl_d | 500 ns | 2 | 1 |
| Repl_e | 500 ns | 3 | 3 |

| Dicationic Conditions -350mV with PIP_2_  V651T/V652T (2-fs) | | | |
| --- | --- | --- | --- |
|  | Simulation Time | Na^+^ Permeation Events | Ca^2+^ Permeation Events |
| Repl_a | 500 ns | 6 | 3 |
| Repl_b | 500 ns | 3 | 6 |
| Repl_c | 500 ns | 3 | 4 |
| Repl_d | 500 ns | 2 | 2 |
| Repl_e | 500 ns | 1 | 2 |

| Monocationic Conditions -350mV with PIP_2_ C623A (2-fs) | | |
| --- | --- | --- |
|  | Simulation Time | Na^+^ Permeation Events |
| Repl_a | ≥250 ns | 0 |
| Repl_b | ≥250 ns | 0 |
| Repl_c | ≥250 ns | 0 |
| Repl_d | ≥250 ns | 0 |
| Repl_e | ≥250 ns | 0 |

Simulations of monocationic solutions under various voltage regimes and with integration time steps as stated in the tables (notes: NR, no hydrophobic gate - HG - restraints used; DW, dewetting of HG observed)

| Monocationic Conditions -700mV with PIP_2_ (4-fs) NR | | |
| --- | --- | --- |
|  | Simulation Time | Na^+^ Permeation Events |
| Repl_a | ≥250 ns | 17 |
| Repl_b | ≥250 ns | 20 |
| Repl_c | ≥250 ns | 1 |
| Repl_d | ≥250 ns | 23 |
| Repl_e | ≥250 ns | 15 |

| Monocationic Conditions -350mV with PIP_2_ (4-fs) NR | | |
| --- | --- | --- |
|  | Simulation Time | Na^+^ Permeation Events |
| Repl_a | ≥250 ns | 9 |
| Repl_b | ≥250 ns | 0 |
| Repl_c | ≥250 ns | 1 |
| Repl_d | ≥250 ns | 0 |
| Repl_e | ≥250 ns | 9 |

| Monocationic Conditions -200mV with PIP_2_ (4-fs) NR, DW | | |
| --- | --- | --- |
|  | Simulation Time | Na^+^ Permeation Events |
| Repl_a | ≥250 ns | 0 |
| Repl_b | ≥250 ns | 0 |
| Repl_c | ≥250 ns | 0 |
| Repl_d | ≥250 ns | 0 |
| Repl_e | ≥250 ns | 0 |

| Monocationic Conditions w/o Voltage with PIP_2_ (4-fs) NR, DW | | |
| --- | --- | --- |
|  | Simulation Time | Na^+^ Permeation Events |
| Repl_a | ≥250 ns | 0 |
| Repl_b | ≥250 ns | 0 |
| Repl_c | ≥250 ns | 0 |
| Repl_d | ≥250 ns | 0 |
| Repl_e | ≥250 ns | 0 |

| Monocationic Conditions +700mV (reversed voltage) with PIP_2_  (4-fs) NR | | |
| --- | --- | --- |
|  | Simulation Time | Na^+^ Permeation Events |
| Repl_a | 330 ns | 57 |
| Repl_b | 330 ns | 10 |
| Repl_c | 330 ns | 54 |
| Repl_d | 330 ns | 25 |
| Repl_e | 330 ns | 6 |

| Monocationic Conditions -700mV with PIP_2_ (2-fs) NR | | |
| --- | --- | --- |
|  | Simulation Time | Ca^+2^ Permeation Events |
| Repl_a | ≥250 ns | 0 |
| Repl_b | ≥250 ns | 1 |
| Repl_c | ≥250 ns | 0 |
| Repl_d | ≥250 ns | 1 |
| Repl_e | ≥250 ns | 0 |

| Monocationic Conditions -200mV with PIP_2_ (2-fs) NR | | |
| --- | --- | --- |
|  | Simulation Time | Ca^+2^ Permeation Events |
| Repl_a | ≥250 ns | 0 |
| Repl_b | ≥250 ns | 0 |
| Repl_c | ≥250 ns | 0 |
| Repl_d | ≥250 ns | 0 |
| Repl_e | ≥250 ns | 0 |

| Monocationic Conditions w/o Voltage with PIP_2_ (2-fs) NR | | |
| --- | --- | --- |
|  | Simulation Time | Ca^+2^ Permeation Events |
| Repl_a | ≥250 ns | 0 |
| Repl_b | ≥250 ns | 0 |
| Repl_c | ≥250 ns | 0 |
| Repl_d | ≥250 ns | 0 |
| Repl_e | ≥250 ns | 0 |

Simulations under -700mV, monocationic solutions and lysosomal membrane composition.

| Monocationic Conditions -700mV with PIP_2_ (4-fs) NR | | |
| --- | --- | --- |
|  | Simulation Time | Na^+^ Permeation Events |
| Repl_a | 330 ns | 11 |
| Repl_b | 330 ns | 2 |
| Repl_c | 330 ns | 2 |
| Repl_d | 330 ns | 6 |
| Repl_e | 330 ns | 13 |

Simulations under various voltage, w/o PIP_2_ and monocationic solutions.

| Monocationic Conditions -700mV w/o PIP_2_ (4-fs) NR | | |
| --- | --- | --- |
|  | Simulation Time | Na^+^ Permeation Events |
| Repl_a | ≥250 ns | 0 |
| Repl_b | ≥250 ns | 0 |
| Repl_c | ≥250 ns | 0 |

| Monocationic Conditions -350mV w/o PIP_2_ (4-fs) NR | | |
| --- | --- | --- |
|  | Simulation Time | Na^+^ Permeation Events |
| Repl_a | ≥250 ns | 0 |
| Repl_b | ≥250 ns | 0 |
| Repl_c | ≥250 ns | 0 |

| Monocationic Conditions -200mV w/o PIP_2_ (4-fs) NR | | |
| --- | --- | --- |
|  | Simulation Time | Na^+^ Permeation Events |
| Repl_a | ≥250 ns | 0 |
| Repl_b | ≥250 ns | 0 |
| Repl_c | ≥250 ns | 0 |

| Monocationic Conditions w/o Voltage and w/o PIP_2_ (4-fs) NR | | |
| --- | --- | --- |
|  | Simulation Time | Na^+^ Permeation Events |
| Repl_a | ≥250 ns | 0 |
| Repl_b | ≥250 ns | 0 |
| Repl_c | ≥250 ns | 0 |
| Repl_d | ≥250 ns | 0 |
| Repl_e | ≥250 ns | 0 |
